# Anemia, Growth Impairment, and Micronutrients Status in Syrian Children Aged 12–60 Months

**DOI:** 10.1155/ijpe/6172527

**Published:** 2025-02-26

**Authors:** Radhee M. Adi, Marie Koenig, Ahmad Alwan, Sami El Khatib, Jürgen Geisel, Stefan Eber, Sahar Chamaa, Rima Obeid

**Affiliations:** ^1^Department of Biochemistry and Microbiology, Faculty of Pharmacy, Damascus University, Damascus, Syria; ^2^Department of Clinical Chemistry and Laboratory Medicine, University Hospital of the Saarland, Homburg/Saar, Germany; ^3^Department of Biomedical Sciences, School of Arts and Sciences, Lebanese International University, Bekaa, Lebanon; ^4^Center for Applied Mathematics and Bioinformatics (CAMB), Gulf University for Science and Technology (GUST), Kuwait City, Kuwait; ^5^Special Praxis for Pediatric Hematology/Oncology, and M1 Private Clinic for Children/Adolescents, Munich, Germany

**Keywords:** anemia, preschool children, stunting, underweight

## Abstract

**Background:** Anemia and growth impairment are major health problems in children under 5 years in countries with ongoing crises.

**Aim**: The aim of this study is to investigate the prevalence of anemia; low *z* scores for height for age, weight for age, and weight for height; and elevated plasma total homocysteine (tHcy) and low folate and vitamin B_12_ concentrations in Syrian children aged 12–60 months.

**Methods**: This cross-sectional study included 344 children of whom physical growth was measured. Blood count and circulating biomarkers of folate and B_12_ and blood count were measured in a subgroup of the children. Data were collected on sociodemographic and contextual factors.

**Results:** Anemia (age-specific cutoff values for hemoglobin according to the World Health Organization) was detected in 24.4% of the children. The height-for-age *z* score was < −2 in 32.0%, the weight-for-age *z* score was < −2 in 16.3%, and the weight-for height *z* sore< −2 in 1.5% of the children. The concentrations of tHcy, folate, and vitamin B_12_ were (median and (10th, 90th percentiles)) 8.4 (5.6, 12.3) *μ*mol/L, 24.0 (9.3, 34.7) nmol/L, and 198 (123, 367) pmol/L, respectively. Anemia and child anthropometric measures were not associated with elevated tHcy, low folate, or low B_12_ concentrations.

**Conclusion:** Anemia and low height- or weight-to-age *z* scores are highly prevalent among Syrian preschool children. Low folate or vitamin B_12_ status was common, but they did not explain these conditions. Future studies may investigate whether early life multinutrient interventions may improve child growth and anemia.

## 1. Introduction

Anemia (defined as low hemoglobin (Hb)), stunting (low height for age), underweight (low weight for age), and wasting (low weight for height) are key indicators of undernutrition and major health problems during early childhood [1, 2]. In 2022, an estimated 45 million children under the age of 5 years were projected to suffer from wasting, while 148 million had stunted growth [3]. Preschool children from low-income countries can develop anemia, stunting, underweight, and wasting because of nutritional deficiencies that are in turn due to socioeconomic and contextual factors [4].

Deficiencies of folate, vitamin B_12_, and iron can contribute to impaired growth and anemia in children [5, 6]. Deficiencies in these micronutrients are alarmingly high in adult people from the Middle East [7–9], but data in children under 5 years of age is scarce. Folate and vitamin B_12_ are used for building the purine and pyrimidine nucleotides needed to synthesize DNA in rapidly growing cells, such as red blood cells in the bone marrow [10, 11]. An elevated plasma concentration of total homocysteine (tHcy) is a marker of folate and/or vitamin B_12_ deficiency [12–14] and may show association with child growth or anemia.

The World Health Organization's (WHO) regional office for the Eastern Mediterranean Region reported 29.6% of Syrian children under five to be stunted in 2020 [15]. The prevalence of anemia among Syrian children (aged 6–59 months) was 32.9% (95% confidence intervals: 14.3, 56.1%) in 2019 as reported in the WHO Global Health Observatory (https://www.who.int/data/gho/data/indicators). The Syrian Arab Republic Nutrition SMART Survey 2019 reported 1.7% of children under five to be wasted and 12.6% to be stunted [16]. However, the prevalence of folate and vitamin B_12_ deficiencies and their associations with child growth and anemia among Syrian children are largely unknown.

The present investigation among Syrian children aged 12–60 months is aimed at studying the prevalence of stunting (height-for-age *z* score < −2), underweight (weight-for-age *z* scores < −2), wasting (weight-for-height *z* score < −2), and anemia and the prevalence of low circulating concentrations of folate and vitamin B_12_ and elevated tHcy. Moreover, we studied the associations between the concentrations of the biomarkers and child growth measures or anemia.

## 2. Subjects and Methods

This explorative cross-sectional study took place between June 2020 and February 2021 at two public health centers located in Damascus (both led by the Syrian Family Planning Association (SFPA)) and one center in Hama (Nutrition Clinic at governmental comprehensive clinics), Syria. The three public centers provide complimentary health care and mainly target people with low socioeconomic status. The inclusion criteria were apparently healthy children aged 12–60 months attending the health centers to receive vaccines, follow-up anthropometric body measurements, or consultation for minor health conditions. The exclusion criteria were congenital abnormalities, hormonal and endocrine disorders that can cause stunting, any diagnosed chronic disease, actual diarrhea, thalassemia trait, fever, and currently manifested infections such as tonsillitis or pneumonia. We did not plan to recruit children under the age of 12 months due to expected difficulties in obtaining consent of the parents and collecting sufficient blood volume.

The study protocol was reviewed and approved by the ethical committee of the College of Pharmacy, University of Damascus, Syria (Approval Number 2019 A/23/7KH). The study was conducted according to the rules documented in the Helsinki Declaration. All parents were informed about the purpose of the study, and they provided a signed written informed consent. Figure S1 shows the study flow diagram.

### 2.1. Child's Anthropometric Measurements

A total of 344 children were recruited and underwent clinical examination in addition to anthropometric measurements conducted by trained professionals at the study centers. The children's weight was measured using a digital scale closest to 0.1 kg. Recumbent length was measured for children younger than 24 months of age. In older children, standing height was measured to the nearest 0.1 cm by using a rigid height board. Body mass index (BMI in kilograms per square meter) was calculated from weight in kilograms and height in square meters. The mid-upper arm circumference (MUAC) and head circumference were measured for the closest 0.1 cm.

Since there are no population-specific growth standards for Syria, the age- and sex-standardized weight, height, and BMI were calculated according to the WHO growth standards (“Length/Height-for-Age” n.d.). These included computed height-for-age *z* score, weight-for-age *z* score, weight-for-height *z* score, and BMI *z* score (separately for girls and boys).

### 2.2. Maternal Information

A standardized questionnaire was used to collect sociodemographic data about the caregivers, living conditions, household income, maternal education level (as the number of school years), maternal weight and height, previous abortions, gravidity, parity, previous diseases or complications during pregnancy with the present child, and duration of the lactation period.

### 2.3. Blood Collection and Measurements

Five-milliliter nonfasting venous blood samples were collected into dry tubes and tubes containing EDTA-K^+^. Blood samples were centrifuged within 30 min after collection, and the serum and plasma were separated and stored at −80°C until analysis. Two novel EDTA-K^+^-plasma samples were shipped on dry ice to the Central Laboratory of the University Hospital of the Saarland, Germany.

Blood count measurements were performed using EDTA-K^+^ blood tubes that were obtained from a subgroup of 329 children. Serum concentrations of folate and vitamin B_12_ were measured in a subgroup of 299 children with a sufficient sample volume using the Abbott chemiluminescent microparticle immunoassay assay (Abbott Diagnostics) on Architect system. The folate assay relies on releasing folate from endogenous folate binding protein and capturing the free folate by paramagnetic microparticles coated with folate binding protein. The signal is detected by a chemiluminescence reaction. The coefficients of variation (CV%) for the folate assay at concentrations of 7.9, 24.5, and 38.5 nmol/L were 3.9%, 3.8%, and 3.6%, respectively.

The B_12_ assay relies on releasing B_12_ in the sample and binding the free B_12_ to intrinsic factor–coated microparticles. The detection is performed by adding a B_12_-acridinium labeled conjugate and measuring the resulting chemiluminescence by optical system. The day-to-day CV% for the vitamin B_12_ assay at concentrations of 182, 314, and 659 pmol/L were 6.2%, 4.0%, and 4.4%, respectively.

Plasma concentrations of tHcy were measured in 335 samples at the Central Laboratory of the Saarland University Hospital, Germany, using a gas chromatography-mass spectrometry method as previously described [17]. The CV% for the tHcy assay at concentrations of 7.6, 13.7, and 27.2 *μ*mol/L were 5.9%, 4.1%, and 7.9%, respectively.

Anemia was defined as Hb concentrations < 10.5, < 11.0, and < 11.5 g/dL for the age groups 6–23 months, 24–59 months, and 60 months, respectively, according to the WHO definition [18]. Children have higher circulating folate and vitamin B_12_ and lower plasma tHcy than adults. Cut-off values for defining folate and B_12_ deficiency in children are not well established and could vary according to the analytical methods [19]. Therefore, we suggestively defined low plasma folate as concentrations < 12.0 nmol/L, low vitamin B_12_ as < 148 pmol/L, and elevated tHcy as concentrations ≥ 8.5*  μ*mol/L (Table 1).

### 2.4. Statistical Analysis

The descriptive results are shown as medians (10th and 90th percentiles) for continuous variables and as absolute (*n*) and relative frequencies (percentage) for categorical variables. The results of Hb and mean corpuscular volume (MCV) are shown as histograms by age and sex of the children (Figures S2 and S3). The one-sample Kolmogorov-Smirnov test and Lilliefors significance correction were used to test the distribution of the continuous variables. In addition, Q-Q plots were generated to additionally evaluate the distribution of the variables. Plasma concentrations of tHcy, folate, and vitamin B_12_ were not normally distributed. Log_10_-transformed data were used for tests that assume a normal distribution.

The one-way analysis of variance (ANOVA) test was used to compare continuous variables (log_10_-transformed) between two independent groups (e.g., boys and girls). The chi-square test was used to compare the distributions of categorical variables among two or more independent groups.

We used binary logistic regression analysis to study the associations between plasma tHcy, folate, and vitamin B_12_ concentrations (high or low concentrations) and height-for-age *z* scores < −2 SD, weight-for-age *z* scores < −2 SD, or anemia (yes vs. no). The definitions of high and low concentrations of the biomarkers were based on the median values of the whole group of children. The crude and fully adjusted regression estimates Exp(*B*) and their 95% confidence intervals (95% CI) are presented for each exposure and outcome of interest.

The covariates were defined prior to approaching the data analysis and consisted of child age (months), sex (M or F), year (2020 or 2021), season (winter, summer, and autumn) of recruitment, study center which also reflect the geographical origin of the children (three recruitment centers), birth order (four categories), pregnancy interval (months), and height (centimeter) and BMI (kg/m^2^) of the mother.

Missing data were not imputed and thus, statistical analysis that adjusted for all covariates was conducted for a lower number of participants. The statistical analyses were conducted using the IBM SPSS Statistics package version 32 (SPSS Inc., Chicago, Illinois, United States). *p* values ≤ 0.05 were considered to indicate statistical significance.

## 3. Results

### 3.1. Child and Maternal Characteristics

The study included 344 children (56.1% girls) with a median (10th, 90th percentiles) age of 30.5 (16.0, 54.0) months (Table 1). Out of the 329 children with blood count results, 84 (24.4%) had anemia with 65 children with mild anemia, 18 with moderate anemia, and 1 child with severe anemia (Table 1). The distributions of Hb and MCV by age and severity of anemia are shown in Figures S4 and S5. The distribution of both Hb and MCV was shifted to the left, suggesting that microcytic rather than macrocytic anemia was the dominant form of anemia in this population.

The median height-for-age *z* scores was −1.54 (10th, 90th percentiles: −2.93, −0.01) and the median weight-for-age *z* scores was −1.03 (10th, 90th percentiles: −2.36, 0.13). Stunting (height-for-age *z* score < −2) was observed in 110 children (32.0%), while 26 children (7.9%) were stunted and had anemia at the same time (Table 1). A low weight-for-age *z* score (< −2) was found in 56 (16.3%) of the children, and a combination of anemia and underweight was observed in 11 children (3.3%). Fifty-one (14.8%) of the children had low *z* scores for height for age and weight for age at the same time (Table 1). A low weight-for-height *z* score was observed in only 1.5% of the children and a low BMI *z* score was found in 1.2%.

Concentrations of tHcy (median (10th, 90th percentiles)) were 8.4 (5.6, 12.3) *μ*mol/L), those of folate were 24.0 (9.3, 34.7) nmol/L, and those of vitamin B_12_ were 198 (123, 367) pmol/L (Table 1). Plasma tHcy concentrations ≥ 8.5*  μ*mol/L were found in 49.6% of the children, plasma  folate concentrations < 12.0 nmol/L were found in 18.4%, and plasma vitamin B_12_ concentrations < 148 pmol/L were found in 23.4%. Both low folate and vitamin B_12_ were found in l0 of 299 children (3.3%).

The concentrations of tHcy, folate, and vitamin B_12_ did not differ according to the sex of the child (Table S1). Sex of the child was not associated with low *z* scores for height for age and *z* scores for weight for age, anemia, or low concentrations of vitamin markers (Table 2). The children who were recruited from the northwest region of the country (Hama) were younger and had a greater prevalence of lower *z* scores for height for age and *z* scores for weight for age than did the children who were recruited from Damascus. The prevalence of decreased *z* scores for height for age and *z* scores for weight for age was highest when the education level of the mother was lowest (Table 2). Of the 62 children who were breastfed at recruitment, 21 (33.9%) had been breastfed for more than 18 months. The use of infant formula was reported for only five of the 62 children. Additional characteristics of the children and their mothers are reported in Table 3 and Table S2, respectively. The frequencies of low BMI-for-age *z* score and weight-for-height *z* scores (*z* scores < −2) were not related to child age, sex, or contextual factors (Table S3).

### 3.2. Child Anthropometrics, Anemia, and Vitamin Markers

The median plasma folate concentration was 14.0 (10th, 90th percentiles: 7.2, 33.0) nmol/L in winter and 29.6 (10th, 90th percentiles: 18.5, 34.4) nmol/L in summer (*p* = 0.004). Elevated concentrations of plasma tHcy were more common in children recruited in summer (June, July, and August) than in those recruited in winter or autumn (Table 2). After adjusting for potential confounders, the logistic regression analyses showed no significant associations between low *z* scores for height for age, *z* scores for weight for age, or anemia in the child and plasma tHcy concentrations ≥ 8.5*  μ*mol/L, plasma folate < 24.0 nmol/L, or plasma vitamin B_12_ concentrations < 199 pmol/L (median values of the whole group) (Table 4).

## 4. Discussion

The present study included generally undernourished children of preschool age with prevalent anemia (30.2%), stunting (32.0%), and underweight (16.3%). The prevalence of anemia and elevated tHcy was highest in the youngest group of children (age between 12 and 23 months) but was not associated with stunting or underweight. Regional differences, a low maternal education level, low household income, and recruitment in the winter months showed associations with growth impairment or abnormal vitamin marker levels in the child. Plasma tHcy, folate, and vitamin B_12_ concentrations suggested that 18%–23% of the children may have insufficient B-vitamin status. Elevated plasma concentrations of tHcy and lowered folate or vitamin B_12_ did not explain anemia, stunting, or underweight after adjusting for confounding factors.

The overall prevalence of anemia in the present study (24.4%) is less than that reported in 2019 by the WHO Global Health Observatory (32.9% (95CI: 14.3, 56.1%)) (https://www.who.int/data/gho/data/indicators) or in Syrian refugee children aged 6–59 months living in Lebanon [21]. The prevalence of stunting in the present study (recruitment time window 2020–2021) is comparable to the 2020 report of the WHO office for the Eastern Mediterranean Region (29.6% of children under five) [15] but was higher compared to Syrian refugee children in Lebanon [22]. This difference could be due to regional differences in food availability or food selection and different practices of using antenatal supplements and fortified infant's formula. The low rate of using infant's formula, extended breastfeeding (up to 18 months and longer), and uncommon use of micronutrient supplements during pregnancy and lactation may explain that anemia and hyperhomocysteinemia were more common in children younger than 24 months.

We found disparities between anemia and abnormal child growth in our study, suggesting different causes of these conditions. In addition, the prevalence of low weight for height and low BMI was only 1.5%, and 1.2%, respectively. Poor diet and especially low intake of animal source foods could explain growth retardation and anemia in children from the present study. In contrast, consumption of macronutrients such as carbohydrates and fats as the main source of calories may lead to weight gain due to higher mass of tissue fat compared to muscles. Previous studies have shown that a low education level and low income are important risk factors for stunting and underweight in children [23, 24]. In addition, low dietary diversity [20] and dependency on seasonal products produced locally [25, 26] could explain our findings of a higher prevalence of low folate (mostly provided through eating fresh vegetables) in children recruited in the rainy months. Common infections in winter, reliance on income from seasonal jobs, and less spending for food due to the need to pay for heat may explain why stunting and underweight were more prevalent in children recruited in the rainy season than in those recruited at other times of the year.

Eliminating anemia and restricted child growth should be considered a priority task in Syrian children. Previous studies suggested that the age < 2 years could be the window of opportunity for interventional studies with multimicronutrients to improve developmental outcomes in the child. In an intervention study, supplementing Indian children with vitamin B_12_ and folic acid for 6 months showed positive effects on several domains of child development (e.g., gross motor and problem solving skills), especially in stunted children, those with a plasma tHcy above 10.0 *μ*mol/L, or those who were younger than 2 years at the end of the study [13]. Supplementing twice the recommended daily intake levels (RDAs) of vitamin B_12_, folic acid, or both (versus placebo) for 2 months in Indian children with anemia did not improve anemia [27], while iron deficiency is considered to be a major cause of childhood anemia in Indian children [27].

The present study has several limitations, such as unavailability of blood samples to measure iron status markers in the child and lack of data on the intake of folate, vitamin B_12_, and iron. Moreover, data linking vitamin deficiencies or anemia to gross motor or neurodevelopmental delay in the child would have added valuable information on potential health outcomes that can be modified by future intervention programs. Finally, collecting blood samples from mothers to study the prevalence of anemia or micronutrient deficiencies could have explained the results.

## 5. Conclusion

In this study among Syrian children aged (12–60 months), we found 24.4% of the children to have mild to moderate anemia, 32.0% to be stunted, and 16.3% to be underweight. Elevated plasma tHcy and decreased folate and vitamin B_12_ concentrations were common but did not show associations with anemia or growth impairment. Impaired child growth was associated with a low mother education level, poverty, a northwestern study location, and the rainy season. Interventions with micronutrients and educational sessions that target low-income families may have large health impact by decreasing the prevalence of micronutrient deficiencies, anemia, stunting, and underweight in children.

## Figures and Tables

**Table 1:** Results of blood analysis and anthropometric measures of all children.

	**n**	**Median (10th and 90th percentiles) or ** **n** ** (%)**
Age (months)	344	30.5 (16.0, 54.0)
Hematocrit (%)	329	35.0 (31.4, 38.3)
Hb (g/dL)	329	11.4 (10.1, 12.6)
Anemia^a^, *n* (%)	329	84 (24.4%)
RBC (10^9^/*μ*L)	329	4.6 (4.1, 5.2)
MCV (fL)	329	77.8 (65.8, 84.2)
MCH (pg)	329	20.9 (25.4, 28.1)
WBC (× 10^3^/*μ*L)	329	8.5 (5.4, 13.1)
Platelets (× 10^6^/*μ*L)	329	315 (204, 466)
Height (cm)	344	86.5 (74.0, 101.5)
Height-for-age *z* score	344	−1.54 (−2.93, −0.01)
Height-for-age *z* score < −2, *n* (%)	344	110 (32.0%)
Anemia and height-for-age *z* score < −2, *n* (%)	329	31 (9.0%)
Weight (kg)	344	11.8 (8.5, 15.7)
Weight-for-age *z* score	344	−1.03 (−2.36, 0.13)
Weight-for-age *z* score < −2, *n* (%)	344	56 (16.3%)
Anemia and weight-for-age *z* score < −2, *n* (%)	329	13 (3.8%)
Height-for-age *z* score < −2 and weight for age *z* score < −2, *n* (%)	344	51 (14.8%)
Weight-for-height *z* scores	344	−0.17 (−1.36, 1.06)
Weight-for-height *z* scores < −2, *n* (%)	344	5 (1.5%)
MUAC (cm)	344	14.9 (13.5, 16.4)
Head circumference (cm)	344	47.5 (44.8, 50.3)
BMI (kg/m^2^)	344	15.5 (14.0, 17.1)
BMI-for-age *z* score	344	−0.09 (−1.31, 1.09)
BMI-for-age *z* score < −2, *n* (%)	344	4 (1.2%)
tHcy (*μ*mol/L)	335	8.4 (5.6, 12.3)
tHcy ≥ 8.5* μ*mol/L	335	166/335 (49.6%)
Folate (nmol/L)	299	24.0 (9.3, 34.7)
Folate < 12.0 nmol/L	299	55/299 (18.4%)
Vitamin B_12_ (pmol/L)	299	198 (123, 367)
Vitamin B_12_ < 148 pmol/L	299	70/299 (23.4%)

Abbreviations: BMI, body mass index; Hb, hemoglobin; MCH, mean corpuscular hemoglobin; MCV, mean corpuscular volume; MUAC, mid-upper arm circumference; RBC, red blood cells; tHcy, total homocysteine; WBC, white blood cells.

^a^Anemia is defined according to the World Health Organization as Hb < 10.5 g/dL for the age group 6–23 months, < 11.0 g/dL for the age group 24–59 months, and < 11.5 g/dL for the age group 60 months.

**Table 2:** Frequency of low anthropometric measures (*z* scores < −2), anemia, and abnormal circulating vitamin markers according to potential influence factors.

	**Outcomes**					
	**Height-for-age ** **z** **score** < −2, **n (%)**	**Weight-for-age ** **z** **scores** < −2, **n (%)**	**Anemia** ^**d**^, **n (%)**	**t** **H** **c** **y** ≥ 8.5** *μ*mol/L**	**F** **o** **l** **a** **t** **e** < 12.0** nmol/L**	**V** **i** **t** **a** **m** **i** **n** **B**_12_ < 148** pmol/L**
Influence factors	The number of children positive for the outcome/total number in the corresponding category (%)					
Sex of the child						
Boys	36/151 (23.8%)	21/151 (13.9%)	42/141 (29.8%)	77/146 (52.7%)	22/129 (17.1%)	33/129 (25.6%)
Girls	61/193 (31.6%)	35/193 (18.1%)	42/188 (22.3%)	89/189 (47.1%)	33/170 (19.4%)	37/170 (21.8%)
*p* ^a^	0.103	0.307	0.128	0.180	0.653	0.078
Child age^b^						
12–23 months	39/111 (35.1%)	20/111 (18.0%)	31/105 (29.5%)	66/108 (61.1%)	17/92 (18.5%)	20/92 (21.7%)
24–39 months	36/117 (30.8%)	22/117 (18.8%)	32/111 (28.8%)	45/112 (40.2%)	22/98 (22.4%)	19/98 (19.4%)
40–60 months	35/116 (30.2%)	14/116 (12.1%)	21/113 (18.6%)	55/115 (47.8%)	16/109 (14.7%)	31/109 (28.4%)
*p* ^a^	0.683	0.316	0.112	**0.007**	0.354	0.277
Study center						
Shaghour Clinic, Damascus	11/48 (22.9%)	5/48 (10.4%)	12/43 (27.9%)	24/44 (54.5%)	1/34 (2.9%)	13/34 (38.2%)
Bab Mousalla Clinic, Damascus	35/189 (18.5%)	15/189 (7.9%)	56/182 (30.8%)	93/187 (49.7%)	6/167 (3.6%)	39/167 (23.4%)
Nutrition Clinic, Hama	64/107 (59.8%)	36/107 (33.6%)	16/104 (15.4%)	49/104 (47.1%)	48/98 (49.0%)	18/98 (18.4%)
*p* ^a^	**< 0.001**	**< 0.001**	**0.023**	0.709	**< 0.001**	0.062
Household income						
Very low	54/108 (50.0%)	32/108 (29.6%)	22/102 (21.6%)	55/103 (53.4%)	32/94 (34.0%)	25/94 (26.6%)
Below average	10/48 (20.8%)	6/48 (12.5%)	17/47 (36.2%)	20/45 (44.4%)	1/40 (2.5%)	9/40 (22.5%)
Average and good	38/144 (26.4%)	16/144 (11.1%)	39/141 (27.7%)	68/144 (47.2%)	17/130 (13.1%)	31/130 (23.8%)
*p* ^a^	**< 0.001**	**< 0.001**	0.168	0.508	**< 0.001**	0.845
Mother education^c^						
0–6 years	43/92 (46.7%)	18/92 (19.6%)	27/87 (31.0%)	52/91 (57.1%)	18/79 (22.8%)	21/79 (26.6%)
> 6–9 years	38/136 (27.9%)	20/136 (14.7%)	28/132 (21.2%)	67/132 (50.8%)	23/120 (19.2%)	31/120 (25.8%)
>9 years	29/111 (26.1%)	18/111 (16.2%)	29/105 (27.6%)	44/107 (41.1%)	12/95 (12.6%)	17/95 (17.9%)
*p* ^a^	**0.014**	0.408	0.378	0.156	0.431	0.424
Season of blood collection						
Winter (December to February)	74/147 (50.3%)	40/147 (27.2%)	30/143 (21.0%)	68/144 (47.2%)	52/133 (39.1%)	26/133 (19.5%)
Summer (June to August)	17/105 (16.2%)	11/105 (10.5%)	29/96 (30.2%)	62/101 (61.4%)	1/80 (1.3%)	25/80 (31.3%)
Autumn (September to November)	19/92 (20.7%)	5/92 (5.4%)	25/90 (27.8%)	36/90 (40.0%)	2/86 (2.3%)	19/86 (22.1%)
*p* ^a^	**< 0.001**	**< 0.001**	0.234	**0.010**	**< 0.001**	0.140
Anemia^d^ in the child						
No (*n* = 245)	80/245 (32.7%)	43/245 (17.6%)	—	116/243 (47.7%)	46/216 (21.3%)	44/216 (20.4%)
Yes (*n* = 84)	26/84 (31.0%)	11/84 (13.1%)	—	43/81 (53.0%)	9/80 (11.3%)	25/80 (31.3%)
*p* ^a^	0.892	0.396	—	0.442	0.063	0.063

*Note:* Information about mother education and household income was collected during an interview at the study center with the mothers or caregivers. *p* values in bold indicate significant between-group differences.

Abbreviations: Hb, hemoglobin; MCV, mean corpuscular volume; tHcy, total homocysteine.

^a^ *p* values for the comparison of the prevalence values between the categories are according to the chi-square test.

^b^Categories of the child age were based on tertiles.

^c^Mother education is shown as number of school years.

^d^Anemia is defined according to the World Health Organization as  grams per deciliter for the age group 6–23 months, < 11.0 g/dL for the age group 24–59 months, and < 11.5 g/dL for the age group 60 months.

**Table 3:** Additional descriptive data of the children (*n* = 344).

**Variable**	**Categories**	**n** ** (%)**
Study center	South of Syria, Damascus	
	Shaghour Clinic	48 (14.0%)
	Bab Musalla Clinic	189 (54.9%)
	Northwest of Syria, Hama	
	Nutrition Clinic	107 (31.1%)

Recruitment year	2020	221 (64.2%)
	2021	123 (35.8%)

Season of recruitment	Winter (December to February)	147 (42.7%)
	Summer (June to August)	105 (30.5%)
	Autumn (September to November)	92 (26.7%)

Birth order of the child	1. Child	94 (27.3%)
	2. Child	87 (25.3%)
	3. Child	80 (23.3%)
	≥ 4. Child	79 (23.0%)
	Missing data	4 (1.2%)

Children who had been breastfed at recruitment	Breastfed up to 12 months	7 (11.3%)
	Breastfed up to 18 months	34 (54.8%)
	Breastfed > 18 months	21 (33.9%)

Infant formula use among children who were breastfed at recruitment	Never used infant formula	57 (91.9%)
	Started infant formula < 6 months	4 (6.5%)
	Started infant formula ≥ 6 months	1 (1.6%)

Duration of breastfeeding in the past in children who were not breastfed at recruitment	Never breastfed	15 (5.3%)
	Up to 6 months	36 (12.8%)
	Up to 12 months	48 (17.0%)
	Up to 18 months	115 (40.8%)
	> 18 months	57 (20.2%)
	Missing data	11 (3.9%)

Infant formula in children who were not breastfed at recruitment	Never used infant formula	192 (68.1%)
	Started < 6 months	64 (22.7%)
	Started ≥ 6 months	13 (4.6%)
	Missing data	13 (4.6%)

**Table 4:** Results of the binary logistic regression analysis of the association between plasma concentrations of homocysteine, folate, or vitamin B_12_ and low height-for-age *z* score, low weight-for-age *z* scores, and anemia in Syrian children.

	**Height-for-age ** **z** **score** < −2			**Weight-for-age ** **z** **scores** < −2			**Anemia** ^**d**^		
	**n** ^**c**^	**Crude**	**Adjusted** ^**a**^	**n** ^**c**^	**Crude**	**Adjusted** ^**a**^	**n** ^**c**^	**Crude**	**Adjusted** ^**a**^
*Plasma tHcy*									
Reference: 3.0–8.4 *μ*mol/L^b^ (total *n* = 169)	58	OR = 1.00	—	27	OR = 1.00	—	43	OR = 1.00	—
8.5–31.9 *μ*mol/L (total *n* = 166)	48	0.77 (0.48, 1.24)	0.82 (0.47, 1.44)	27	0.91 (0.50, 1.65)	0.93 (0.48, 1.83)	58	1.26 (0.76–2.10)	1.13 (0.65–1.96)
*Plasma folate*									
Reference: 20.0–38.3 nmol/L^b^ (total *n* = 150)	28	OR = 1.00	—	13	OR = 1.00	—	51	OR = 1.00	—
4.3–23.8 nmol/L (total *n* = 149)	70	3.99 (2.31, 6.86)	1.44 (0.70, 2.97)	37	3.53 (1.75, 7.13)	1.35 (0.53, 3.44)	41	0.52 (0.24–1.12)	0.81 (0.31–2.14)
*Plasma vitamin B_12_*									
Reference: 199–663 pmol/L^b^ (total *n* = 151)	53	OR = 1.00	—	32	OR = 1.00	—	44	OR = 1.00	—
37–198 pmol/L (total *n* = 148)	45	0.82 (0.50, 1.34)	0.93 (0.51, 1.70)	18	0.48 (0.25, 0.92)	0.50 (0.24, 1.04)	48	1.66 (0.92–3.01)	1.78 (0.93–3.43)

*Note:* The odds ratio (OR) and 95% confidence intervals (95% CI) of children with high plasma homocysteine or low plasma folate or B_12_ to have one of the study outcomes (height-for-age *z* score < −2, weight-for-age *z* scores < −2, or anemia).

Abbreviations: BMI, body mass index; Hb, hemoglobin; tHcy, total homocysteine.

^a^Fully adjusted model included the following covariates: age and sex of the child, year and season of recruitment, study center, birth order, pregnancy interval in months, and BMI and height of the mother.

^b^Categories of high and low concentrations of the markers were based on the corresponding median values (≥ 8.5 *μ*mol/L for tHcy, < 24.0 nmol/L for plasma folate, and < 199 pmol/L for plasma vitamin B_12_).

^c^Number of children positive for the outcome.

^d^Anemia is defined according to the World Health Organization as Hb < 10.5 g/dL for the age group 6–23 months, < 11.0 g/dL for the age group 24–59 months, and < 11.5 g/dL for the age group 60 months.

## Data Availability

Data is available in an aggregated form from the corresponding author upon reasonable request.

## Nomenclature

BMI: body mass index
Hb: hemoglobin
CV%: coefficients of variation
MCH: mean corpuscular hemoglobin
MCV: mean corpuscular volume
MUAC: mid-upper arm circumference
RBC: red blood cell
tHcy: total homocysteine
WBC: white blood cell
WHO: World Health Organization

## Supporting Information

Supporting Information Additional supporting information can be found online in the Supporting Information section. Table S1: Results of blood count analysis, anthropometric measures, and vitamin markers according to sex. Table S2: Characteristics of the mothers. Table S3: Frequency of low BMI-for-age *z* score and weight-for-height *z* scores (*z* scores < −2) according to potential influence factors. Figure S1: Study flow diagram. Figure S2: Histogram of hemoglobin in 329 Syrian children (*n* = 141 boys and 188 girls) by age and sex groups. Figure S3: Histogram of mean corpuscular volume in 329 Syrian children (*n* = 141 boys and 188 girls) by age and sex groups. Figure S4: Scatter plot of the relationship between hemoglobin and child age according to subgroups of anemia severity. The severity of anemia was defined according to the World Health Organization (World Health Organization (2024). Guideline on hemoglobin cutoffs to define anemia in individuals and populations. World Health Organization. https://iris.who.int/handle/10665/376196. License: CC BY-NC-SA 3.0 IGO). Figure S5: Scatter plot of the relationship between mean corpuscular volume and child age according to subgroups of anemia severity. The severity of anemia was defined according to the World Health Organization (World Health Organization (2024). Guideline on hemoglobin cutoffs to define anemia in individuals and populations. World Health Organization. https://iris.who.int/handle/10665/376196. License: CC BY-NC-SA 3.0 IGO).
