# Supplementary material for: Anemia, Growth Impairment, and Micronutrients Status in Syrian Children Aged 12–60 Months
Source: Int J Pediatr. 2025 Feb 26;2025:6172527. doi: 10.1155/ijpe/6172527 (PMC11985240; doi:10.1155/ijpe/6172527)
Supplement: Supporting Information — Additional supporting information can be found online in the Supporting Information section. Table S1: Results of blood count analysis, anthropometric measures, and vitamin markers according to sex. Table S2: Characteristics of the mothers. Table S3: Frequency of low BMI-for-age z score and weight-for-height z scores (z scores < −2) according to potential influence factors. Figure S1: Study flow diagram. Figure S2: Histogram of hemoglobin in 329 Syrian children (n = 141 boys and 188 girls) by age and sex groups. Figure S3: Histogram of mean corpuscular volume in 329 Syrian children (n = 141 boys and 188 girls) by age and sex groups. Figure S4: Scatter plot of the relationship between hemoglobin and child age according to subgroups of anemia severity. The severity of anemia was defined according to the World Health Organization (World Health Organization (2024). Guideline on hemoglobin cutoffs to define anemia in individuals and populations. World Health Organization. https://iris.who.int/handle/10665/376196. License: CC BY-NC-SA 3.0 IGO). Figure S5: Scatter plot of the relationship between mean corpuscular volume and child age according to subgroups of anemia severity. The severity of anemia was defined according to the World Health Organization (World Health Organization (2024). Guideline on hemoglobin cutoffs to define anemia in individuals and populations. World Health Organization. https://iris.who.int/handle/10665/376196. License: CC BY-NC-SA 3.0 IGO). [file 6172527.f1.zip › R2 Supplementary Tables Final.docx]

**Anemia, growth impairment, and micronutrients status in Syrian children aged 12-60 months**

**Supplementary Tables**

| Supplementary Table 1. Results of blood count analysis, anthropometric measures, and vitamin markers according to sex. | | | | | | |
| --- | --- | --- | --- | --- | --- | --- |
|  | n | Boys |  | n | Girls | p^1^ |
| Age, months | 151 | 33.0 (15.2, 54.0) |  | 193 | 29 (16.0, 54.0) | 0.791 |
| Hematocrit, % | 141 | 34.8 (31.4, 38.6) |  | 188 | 35.1 (31.3, 38.2) | 0.903 |
| Hb, g/dl | 141 | 11.3 (10.0, 12.6) |  | 188 | 11.4 (10.2, 12.7) | 0.594 |
| Anemia^3^, n (%) | 141 | 42 (29.8%) |  | 188 | 42 (22.3%) | 0.128^2^ |
| RBC, 10^9^/µl | 141 | 4.6 (4.1, 5.1) |  | 188 | 4.6 (4.1, 5.2) | 0.771 |
| MCV, fL | 141 | 77.7 (63.1, 84.4) |  | 188 | 77.9 (66.5, 84.2) | 0.754 |
| WBC, ×10^3^ /µL | 141 | 8.5 (5.5, 12.5) |  | 188 | 8.6 (5.2, 13.5) | 0.116 |
| Platelets, ×10^6^/µL | 141 | 308 (204, 427) |  | 188 | 315 (204, 475) | 0.175 |
| Height, cm | 151 | 88.4 (75.0, 103.0) |  | 193 | 85.0 (72.4, 100.5) | 0.009 |
| Height-for-age z score | 151 | -1.35 (-2.83, 0.10) |  | 193 | -1.59 (-3.22, -0.14) | 0.008 |
| Height-for-age z score < -2, n (%) | 151 | 36 (23.8%) |  | 193 | 61 (31.6%) | 0.103^2^ |
| Anemia and Height-for-age z score < -2, n (%) | 141 | 13 (8.6%) |  | 188 | 18 (9.3%) | 0.999^2^ |
| Weight, kg | 151 | 12.2 (9.0, 16.3) |  | 193 | 11.0 (8.2, 15.4) | 0.001 |
| Weight-for-age z score | 151 | -0.89 (-2.32, 0.38) |  | 193 | -1.12 (-2.42, -0.10) | 0.011 |
| Weight-for-age z score < -2, n (%) | 151 | 21 (13.9%) |  | 193 | 35 (18.1%) | 0.307^2^ |
| Anemia and weight-for-age z score < -2, n (%) | 141 | 5 (3.3%) |  | 188 | 8 (4.1%) | 0.784^2^ |
| Height-for-age z score < -2 and weight for age z score < -2, n (%) | 151 | 19 (12.6%) |  | 193 | 32 (16.6%) | 0.391^2^ |
| Weight-for-height z scores |  | -0.10 (-1.46, 1.12) |  |  | -0.20 (-1.33, 0.85) | 0.397 |
| MUAC, cm | 151 | 15.0 (13.5, 16.6) |  | 193 | 14.9 (13.4, 16.2) | 0.068 |
| Head circumference, cm | 151 | 48.0 (45.5, 50.5) |  | 193 | 47.0 (44.5, 49.5) | <0.001 |
| BMI, kg/m^2^ | 151 | 15.7 (14.2, 17.5) |  | 193 | 15.4 (13.9, 16.8) | 0.010 |
| BMI-for-age z score | 151 | -0.078 (-1.37, 1.19) |  | 193 | -0.10 (-1.29, 0.96) | 0.493 |
| tHcy, µmol/L | 146 | 8.6 (5.8, 12.1) |  | 189 | 8.1 (5.4, 12.4) | 0.144 |
| tHcy ≥ 8.5 µmol/L, n(%) |  | 77/146 (52.7%) |  |  | 89/189 (47.1%) | 0.180^2^ |
| Folate, nmol/L | 129 | 22.9 (8.8, 34.0) |  | 170 | 24.9 (9.3, 34.9) | 0.748 |
| Folate < 12.0 nmol/L, n(%) |  | 22/129 (17.1%) |  |  | 33/170 (19.4%) | 0.653^2^ |
| Vitamin B12, pmol/L | 129 | 195 (114, 352) |  | 170 | 209 (125, 408) | 0.078 |
| Vitamin B12 < 148 pmol/L, n(%) |  | 33/129 (25.6%) |  |  | 37/170 (21.8%) | 0.491^2^ |
| Data are median (10^th^, 90^th^ percentiles) or n (%).  ^1^ p values for the difference in continuous variables between boys and girls are according to ANOVA test was applied using either the z-scores or the log-transformed values.  ^2^ Categorical variables are compared between boys and girls using the chi-square test.  ^3^ Anemia is defined according to the World Health Organization as Hb < 10.5 g/dl for the age group 6-23 months, < 11.0 g/dl for the age group 24-59 months, and < 11.5 g/dl for the age group 60 months.  BMI, body mass index; Hb, hemoglobin; MCV, mean corpuscular volume; MUAC, Mid-Upper Arm Circumference; RBC, red blood cells; tHcy, total homocysteine; WBC, white blood cells. | | | | | | |

| Supplementary Table 2. Characteristics of the mothers. | | |
| --- | --- | --- |
| Mother variables | Median (10^th^, 90^th^ percentiles) | |
| Age, years (n = 337) | 29 (22, 38) | |
| Weight, kg (n = 344) | 60.0 (50.0, 81.5) | |
| Height, cm (n = 344) | 158 (150, 165) | |
| BMI, kg/m^2^ (n = 344) | 24.3 (20.4, 31.7) | |
| Pregnancy intervals^1^, months (n = 323) |  | |
| First pregnancy | 91 (26.5%) | |
| 2-12 months | 32 (9.3%) | |
| 13-24 months | 60 (17.4%) | |
| > 24 months | 140 (40.7%) | |
|  | Category | n (%) |
| Delivery form | Cesarean section  Natural birth  missing data | 186 (54.1%)  138 (40.1%)  20 (5.8%) |
| Previous pregnancy termination/abortion | 0  1  2  ≥3  missing data | 240 (69.8%)  44 (12.8%)  24 (7.0%)  25 (7.4%)  11 (3.2%) |
| Mother education level (n school years) | 0 to 6 years  >6 to 9 years  >9 to 12 years  >12 years  missing data | 92 (26.8%)  136 (39.5%)  66 (19.2%)  45 (13.1%)  5 (1.5%) |
| Income (self-reported) | Very low  Below average  Average and good  missing data | 108 (31.4%)  48 (14.0%)  144 (41.9%)  44 (12.8%) |
| BMI, Body mass index.  ^1^ intervals between two completes pregnancies (miscarriages were not considered). | | |

| Supplementary Table 3. Frequency of low BMI-for-age z score and weight-for-height z scores (z scores < -2) according to potential influence factors. | | |
| --- | --- | --- |
|  | **BMI-for-age z score < -2, n (%)** | **Weight-for-height z scores < -2, n (%)** |
| **Sex of the child** |  |  |
| Boys | 3/151 | 3/151 |
| Girls | 1/193 | 2/193 |
| **p** | 0.323 | 0.657 |
| **Child age** |  |  |
| 12-23 months | 1/111 | 2/111 |
| 24-39 months | 1/117 | 1/117 |
| 40-60 months | 2/116 | 2/116 |
| p | 0.560 | 0.968 |
| **Study center** |  |  |
| Shaghour Clinic, Damascus | 0/48 | 0/48 |
| Bab Mousalla Clinic, Damascus | 3/189 | 3/189 |
| Nutrition Clinic, Hama | 1/107 | 2/107 |
| p | 0.808 | 0.428 |
| **Household income** |  |  |
| Very low | 0/108 | 0/108 |
| Below average | 0/48 | 0/48 |
| Average and good | 4/144 | 5/144 |
| p | **0.052** | **0.029** |
| **Mother education** |  |  |
| 0 to 6 years | 1/92 | 2/92 |
| > 6 to 9 years | 0/136 | 0/136 |
| > 9 years | 3/222 | 3/111 |
| p | **0.099** | 0.341 |
| **Season of blood collection** |  |  |
| Winter (December to February) | 1/147 | 2/147 |
| Summer (June to August ) | 1/105 | 1/105 |
| Autumn (September to November) | 2/92 | 2/92 |
| p | **0.314** | **0.660** |
| p values are according to the chi-square test.  Information about mother education and household income were collected during an interview at the study center with the mothers or caregivers.  Hb, hemoglobin; MCV, mean corpuscular volume. | | |
