# Supplementary figures and images for: Anemia, Growth Impairment, and Micronutrients Status in Syrian Children Aged 12–60 Months
Source: Int J Pediatr. 2025 Feb 26;2025:6172527. doi: 10.1155/ijpe/6172527 (PMC11985240; doi:10.1155/ijpe/6172527)

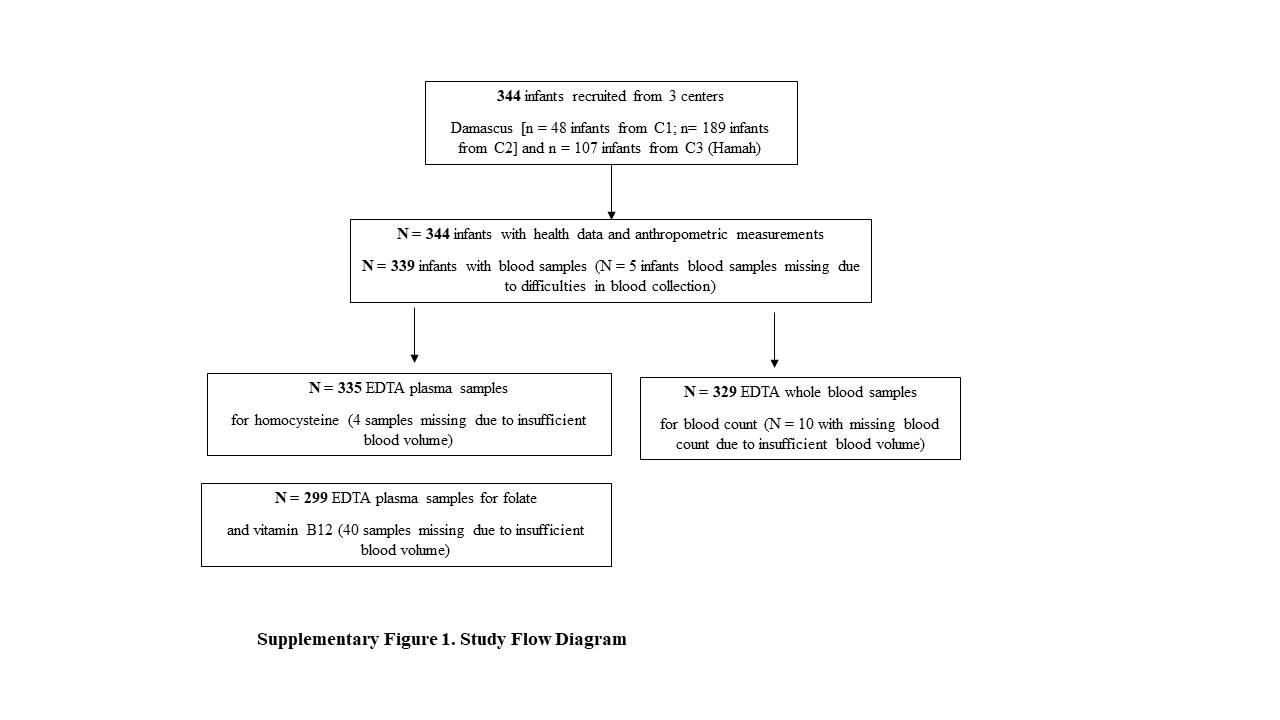

Supplement: Supporting Information — Additional supporting information can be found online in the Supporting Information section. Table S1: Results of blood count analysis, anthropometric measures, and vitamin markers according to sex. Table S2: Characteristics of the mothers. Table S3: Frequency of low BMI-for-age z score and weight-for-height z scores (z scores < −2) according to potential influence factors. Figure S1: Study flow diagram. Figure S2: Histogram of hemoglobin in 329 Syrian children (n = 141 boys and 188 girls) by age and sex groups. Figure S3: Histogram of mean corpuscular volume in 329 Syrian children (n = 141 boys and 188 girls) by age and sex groups. Figure S4: Scatter plot of the relationship between hemoglobin and child age according to subgroups of anemia severity. The severity of anemia was defined according to the World Health Organization (World Health Organization (2024). Guideline on hemoglobin cutoffs to define anemia in individuals and populations. World Health Organization. https://iris.who.int/handle/10665/376196. License: CC BY-NC-SA 3.0 IGO). Figure S5: Scatter plot of the relationship between mean corpuscular volume and child age according to subgroups of anemia severity. The severity of anemia was defined according to the World Health Organization (World Health Organization (2024). Guideline on hemoglobin cutoffs to define anemia in individuals and populations. World Health Organization. https://iris.who.int/handle/10665/376196. License: CC BY-NC-SA 3.0 IGO). [file 6172527.f1.zip › Folie1.JPG]

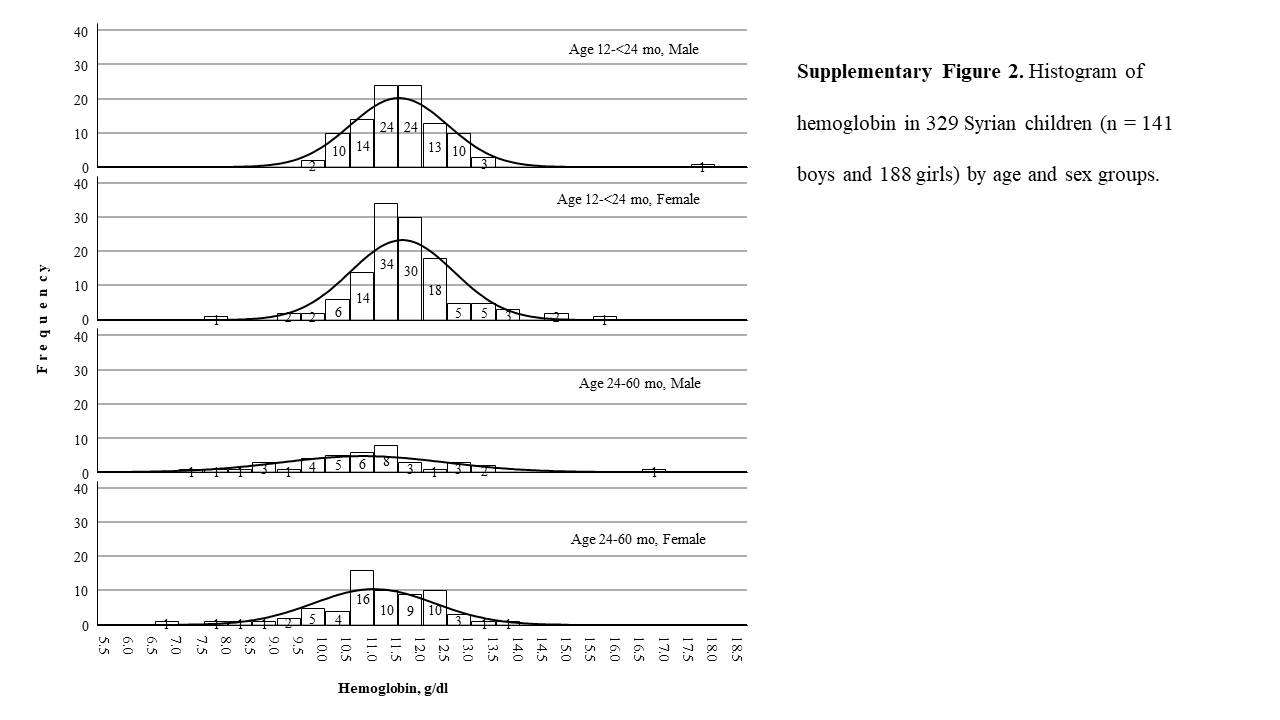

Supplement: Supporting Information — Additional supporting information can be found online in the Supporting Information section. Table S1: Results of blood count analysis, anthropometric measures, and vitamin markers according to sex. Table S2: Characteristics of the mothers. Table S3: Frequency of low BMI-for-age z score and weight-for-height z scores (z scores < −2) according to potential influence factors. Figure S1: Study flow diagram. Figure S2: Histogram of hemoglobin in 329 Syrian children (n = 141 boys and 188 girls) by age and sex groups. Figure S3: Histogram of mean corpuscular volume in 329 Syrian children (n = 141 boys and 188 girls) by age and sex groups. Figure S4: Scatter plot of the relationship between hemoglobin and child age according to subgroups of anemia severity. The severity of anemia was defined according to the World Health Organization (World Health Organization (2024). Guideline on hemoglobin cutoffs to define anemia in individuals and populations. World Health Organization. https://iris.who.int/handle/10665/376196. License: CC BY-NC-SA 3.0 IGO). Figure S5: Scatter plot of the relationship between mean corpuscular volume and child age according to subgroups of anemia severity. The severity of anemia was defined according to the World Health Organization (World Health Organization (2024). Guideline on hemoglobin cutoffs to define anemia in individuals and populations. World Health Organization. https://iris.who.int/handle/10665/376196. License: CC BY-NC-SA 3.0 IGO). [file 6172527.f1.zip › Folie2.JPG]

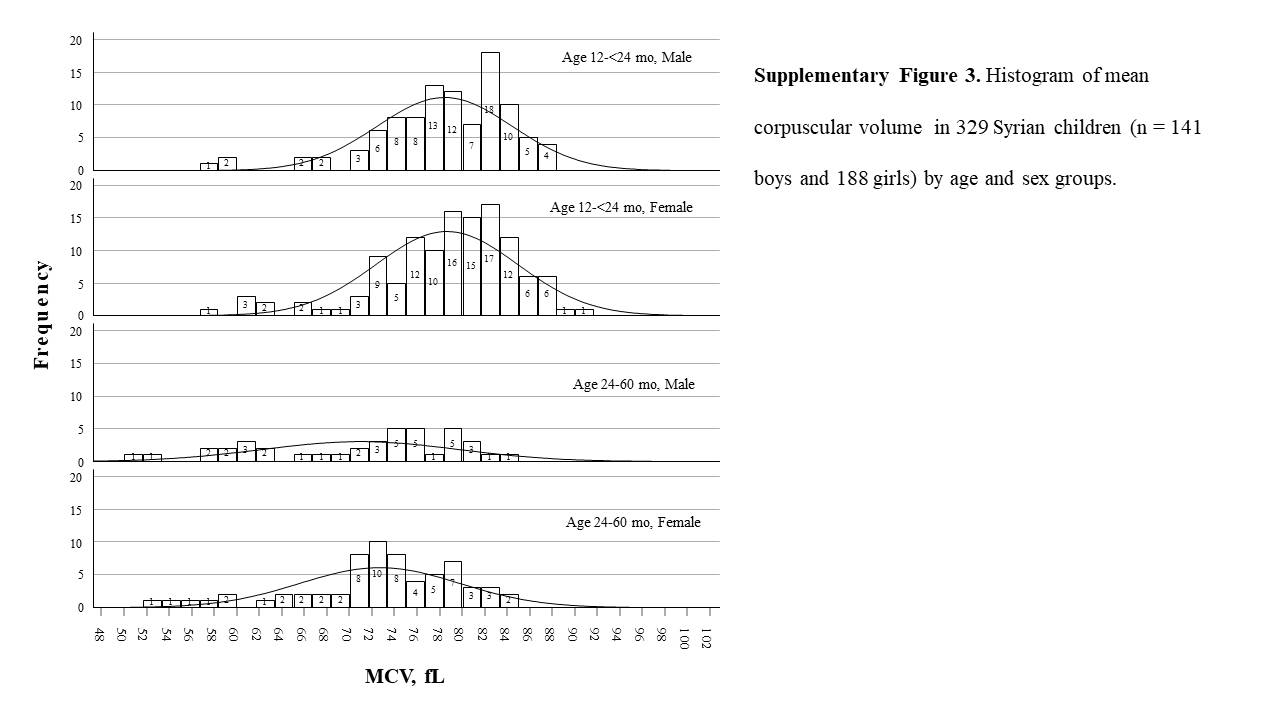

Supplement: Supporting Information — Additional supporting information can be found online in the Supporting Information section. Table S1: Results of blood count analysis, anthropometric measures, and vitamin markers according to sex. Table S2: Characteristics of the mothers. Table S3: Frequency of low BMI-for-age z score and weight-for-height z scores (z scores < −2) according to potential influence factors. Figure S1: Study flow diagram. Figure S2: Histogram of hemoglobin in 329 Syrian children (n = 141 boys and 188 girls) by age and sex groups. Figure S3: Histogram of mean corpuscular volume in 329 Syrian children (n = 141 boys and 188 girls) by age and sex groups. Figure S4: Scatter plot of the relationship between hemoglobin and child age according to subgroups of anemia severity. The severity of anemia was defined according to the World Health Organization (World Health Organization (2024). Guideline on hemoglobin cutoffs to define anemia in individuals and populations. World Health Organization. https://iris.who.int/handle/10665/376196. License: CC BY-NC-SA 3.0 IGO). Figure S5: Scatter plot of the relationship between mean corpuscular volume and child age according to subgroups of anemia severity. The severity of anemia was defined according to the World Health Organization (World Health Organization (2024). Guideline on hemoglobin cutoffs to define anemia in individuals and populations. World Health Organization. https://iris.who.int/handle/10665/376196. License: CC BY-NC-SA 3.0 IGO). [file 6172527.f1.zip › Folie3.JPG]

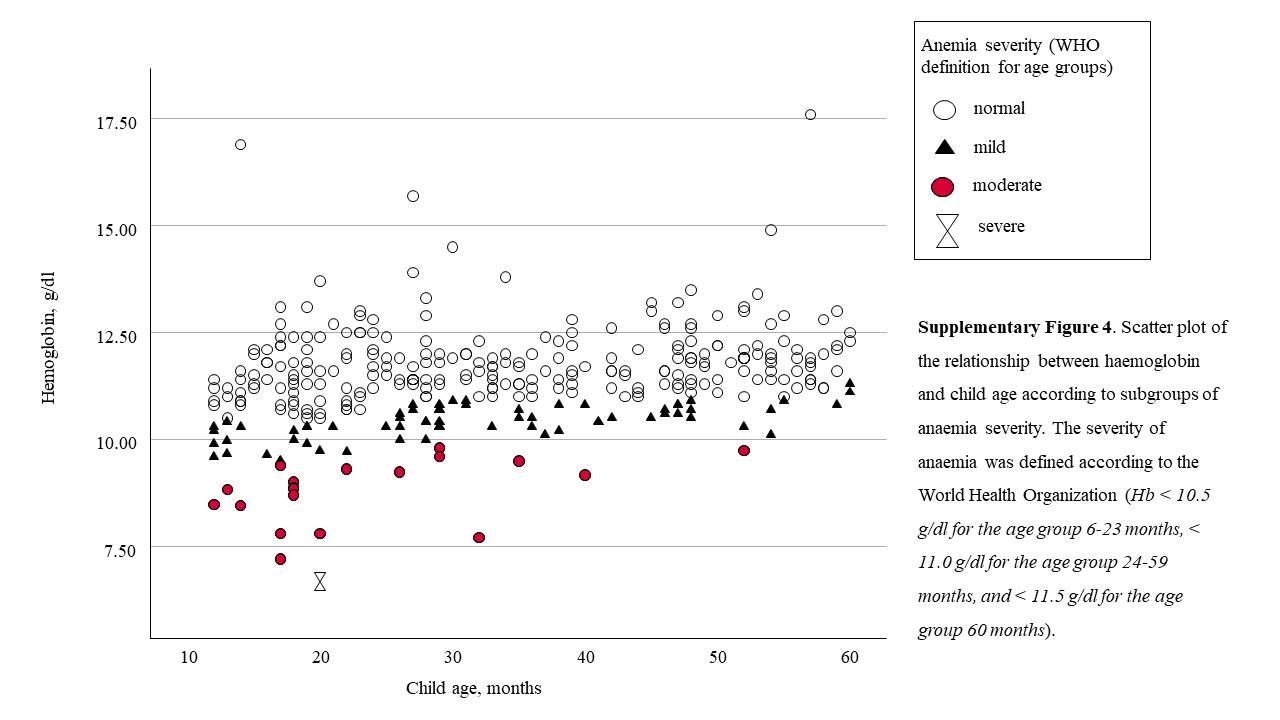

Supplement: Supporting Information — Additional supporting information can be found online in the Supporting Information section. Table S1: Results of blood count analysis, anthropometric measures, and vitamin markers according to sex. Table S2: Characteristics of the mothers. Table S3: Frequency of low BMI-for-age z score and weight-for-height z scores (z scores < −2) according to potential influence factors. Figure S1: Study flow diagram. Figure S2: Histogram of hemoglobin in 329 Syrian children (n = 141 boys and 188 girls) by age and sex groups. Figure S3: Histogram of mean corpuscular volume in 329 Syrian children (n = 141 boys and 188 girls) by age and sex groups. Figure S4: Scatter plot of the relationship between hemoglobin and child age according to subgroups of anemia severity. The severity of anemia was defined according to the World Health Organization (World Health Organization (2024). Guideline on hemoglobin cutoffs to define anemia in individuals and populations. World Health Organization. https://iris.who.int/handle/10665/376196. License: CC BY-NC-SA 3.0 IGO). Figure S5: Scatter plot of the relationship between mean corpuscular volume and child age according to subgroups of anemia severity. The severity of anemia was defined according to the World Health Organization (World Health Organization (2024). Guideline on hemoglobin cutoffs to define anemia in individuals and populations. World Health Organization. https://iris.who.int/handle/10665/376196. License: CC BY-NC-SA 3.0 IGO). [file 6172527.f1.zip › Folie4.JPG]

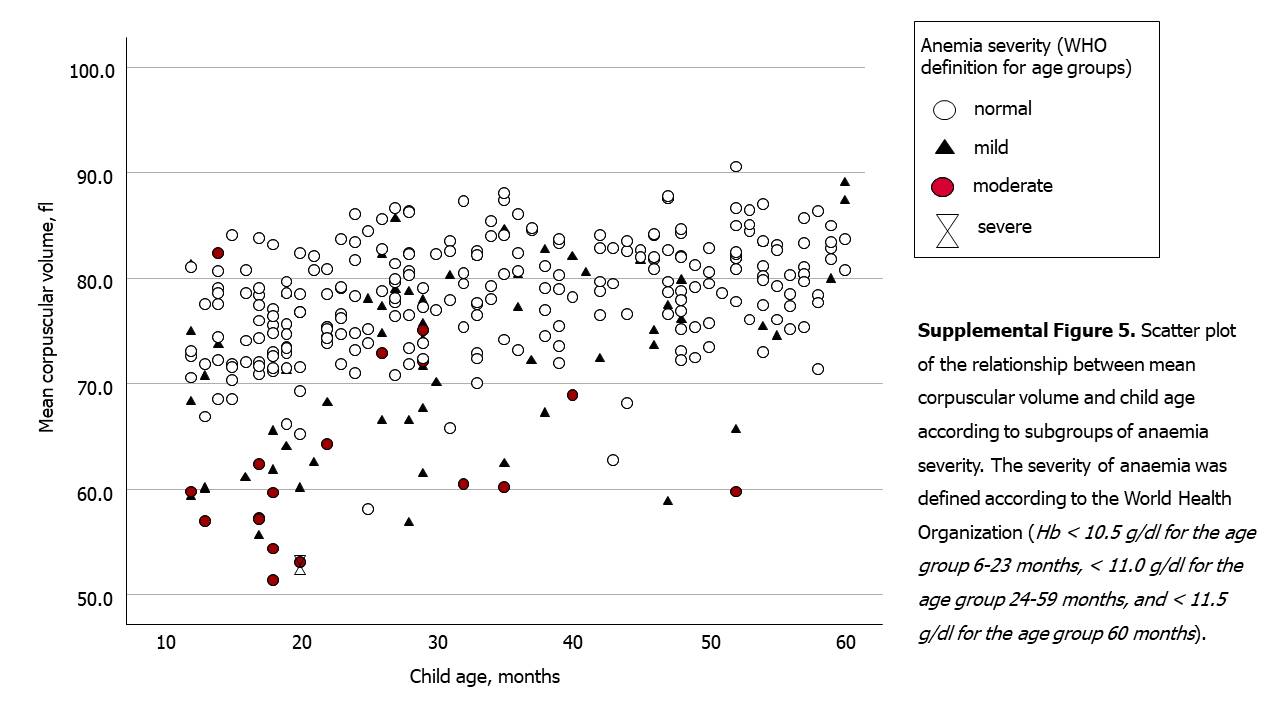

Supplement: Supporting Information — Additional supporting information can be found online in the Supporting Information section. Table S1: Results of blood count analysis, anthropometric measures, and vitamin markers according to sex. Table S2: Characteristics of the mothers. Table S3: Frequency of low BMI-for-age z score and weight-for-height z scores (z scores < −2) according to potential influence factors. Figure S1: Study flow diagram. Figure S2: Histogram of hemoglobin in 329 Syrian children (n = 141 boys and 188 girls) by age and sex groups. Figure S3: Histogram of mean corpuscular volume in 329 Syrian children (n = 141 boys and 188 girls) by age and sex groups. Figure S4: Scatter plot of the relationship between hemoglobin and child age according to subgroups of anemia severity. The severity of anemia was defined according to the World Health Organization (World Health Organization (2024). Guideline on hemoglobin cutoffs to define anemia in individuals and populations. World Health Organization. https://iris.who.int/handle/10665/376196. License: CC BY-NC-SA 3.0 IGO). Figure S5: Scatter plot of the relationship between mean corpuscular volume and child age according to subgroups of anemia severity. The severity of anemia was defined according to the World Health Organization (World Health Organization (2024). Guideline on hemoglobin cutoffs to define anemia in individuals and populations. World Health Organization. https://iris.who.int/handle/10665/376196. License: CC BY-NC-SA 3.0 IGO). [file 6172527.f1.zip › Folie5.JPG]
